# Comparison of prostate delineation on multimodality imaging for MR-guided radiotherapy

**DOI:** 10.1259/bjr.20180948

**Published:** 2019-01-11

**Authors:** Angela U Pathmanathan, Helen A McNair, Maria A Schmidt, Douglas H Brand, Louise Delacroix, Cynthia L Eccles, Alexandra Gordon, Trina Herbert, Nicholas J van As, Robert A Huddart, Alison C Tree

**Affiliations:** 1 The Royal Marsden Hospital NHS Foundation Trust, Downs Road, Sutton, United Kingdom; 2 The Institute of Cancer Research, 15 Cotswold Road, Sutton, United Kingdom

## Abstract

**Objective:**

With increasing incorporation of MRI in radiotherapy, we investigate two MRI sequences for prostate delineation in radiographer-led image guidance.

**Methods:**

Five therapeutic radiographers contoured the prostate individually on CT, *T*_2_ weighted (*T*_2_W) and *T*_2_* weighted (*T*_2_*W) imaging for 10 patients. Contours were analysed with Monaco ADMIRE (research v. 2.0) to assess interobserver variability and accuracy by comparison with a gold standard clinician contour. Observers recorded time taken for contouring and scored image quality and confidence in contouring.

**Results:**

There is good agreement when comparing radiographer contours to the gold-standard for all three imaging types with Dice similarity co-efficient 0.91–0.94, Cohen’s κ 0.85–0.91, Hausdorff distance 4.6–7.6 mm and mean distance between contours 0.9–1.2 mm. In addition, there is good concordance between radiographers across all imaging modalities. Both *T*_2_W and *T*_2_*W MRI show reduced interobserver variability and improved accuracy compared to CT, this was statistically significant for *T*_2_*W imaging compared to CT across all four comparison metrics. Comparing MRI sequences reveals significantly reduced interobserver variability and significantly improved accuracy on *T*_2_*W compared to *T*_2_W MRI for DSC and Cohen’s κ. Both MRI sequences scored significantly higher compared to CT for image quality and confidence in contouring, particularly *T*_2_*W. This was also reflected in the shorter time for contouring, measuring 15.4, 9.6 and 9.8 min for CT, *T*_2_W and *T*_2_*W MRI respectively.

**Conclusion:**

Therapeutic radiographer prostate contours are more accurate, show less interobserver variability and are more confidently and quickly outlined on MRI compared to CT, particularly using *T*_2_*W MRI.

**Advances in knowledge:**

Our work is relevant for MRI sequence choice and development of the roles of the interprofessional team in the advancement of MRI-guided radiotherapy.

## Introduction

MRI provides a number of benefits in radiotherapy (RT) of the prostate, including improved soft tissue resolution for prostate and organs at risk delineation and multiparametric imaging for intraprostatic lesion identification and response assessment. There has been increasing interest in MR-guided systems^1,2^ to encompass these advantages and permit intrafractional imaging without additional radiation exposure.^3^ With variability in prostate and seminal vesicles contouring dependent on the sequence used,^4^ sequence optimisation is vital to maintain accuracy. In addition, prostate delineation must be completed in a timely manner when used in an online or real-time adaptive setting.

Dedicated MRI sequences can enhance the signal void of fiducials,^5,6^ required for accurate MRI and CT fusion^7^ and position verification prior to treatment. One such sequence, *T*_2_*-weighted (*T*_2_*W) MRI, uses multiple echo times^8^ resulting in a more defined prostate capsule as well as a reliable depiction of fiducials; geometric accuracy and clinician contouring consistency on this type of sequence has previously been assessed.^9^

With relative unfamiliarity of MRI compared to CT, MRI must be introduced carefully into the RT planning process involving all members of the interprofessional team, together with appropriate training.^10^ Therapeutic radiographers at our centre are experienced in reviewing the prostate position on cone beam CT (CBCT) for image guidance prior to treatment delivery. RT services benefit from the expanded role of therapeutic radiographers including radiographer-led delineation of the target or organs at risk^11^ which can shorten the treatment planning process.^12^ At our institution, following a training programme, specialised therapeutic radiographers outline the prostate and seminal vesicles on the RT planning CT, prior to clinician review and final approval.

However, with the emergence of new technologies, this must be extended to prostate identification on MRI for MRI-guided RT. With the installation of the Elekta MR-Linac^1^ at our centre and treatment of our first patient in September 2018, we wish to extend the therapeutic radiographer role to include delineation of the prostate on MRI. This will be particularly relevant for adaptive online replanning where recontouring and intrafraction monitoring of the target is required.

With RT workflow changing, the work we present here addresses an important area which has not been well studied to date. Despite the evolving role of therapeutic radiographers, to our knowledge there are no publications demonstrating the accuracy and consistency of radiographer-derived contours which is an essential part of treatment quality assurance. Our study assesses the interobserver variability and accuracy of prostate delineation by therapeutic radiographers using three imaging types; CT, *T*_2_ weighted (*T*_2_W) and *T*_2_*W MRI.

## Methods and Materials

### Patient population

The patient population and image acquisition have previously been described.^9^ 10 patients receiving treatment within the Prostate Advances in Comparative Evidence (PACE) trial (NCT01584258) at the Royal Marsden Hospital NHS Foundation Trust, Sutton, had RT planning CT and MRI scans acquired on the same day. The PACE trial has two parallel randomisations; PACE A randomises between prostatectomy or stereotactic body radiotherapy (SBRT) to a dose of 36.25 Gy in five fractions, and PACE B randomised patients between SBRT or conventionally fractionated RT, either 62 Gy in 20 fractions or 78 Gy in 39 fractions. Patients do not receive androgen deprivation therapy.

### Image acquisition

**Figure 1.**
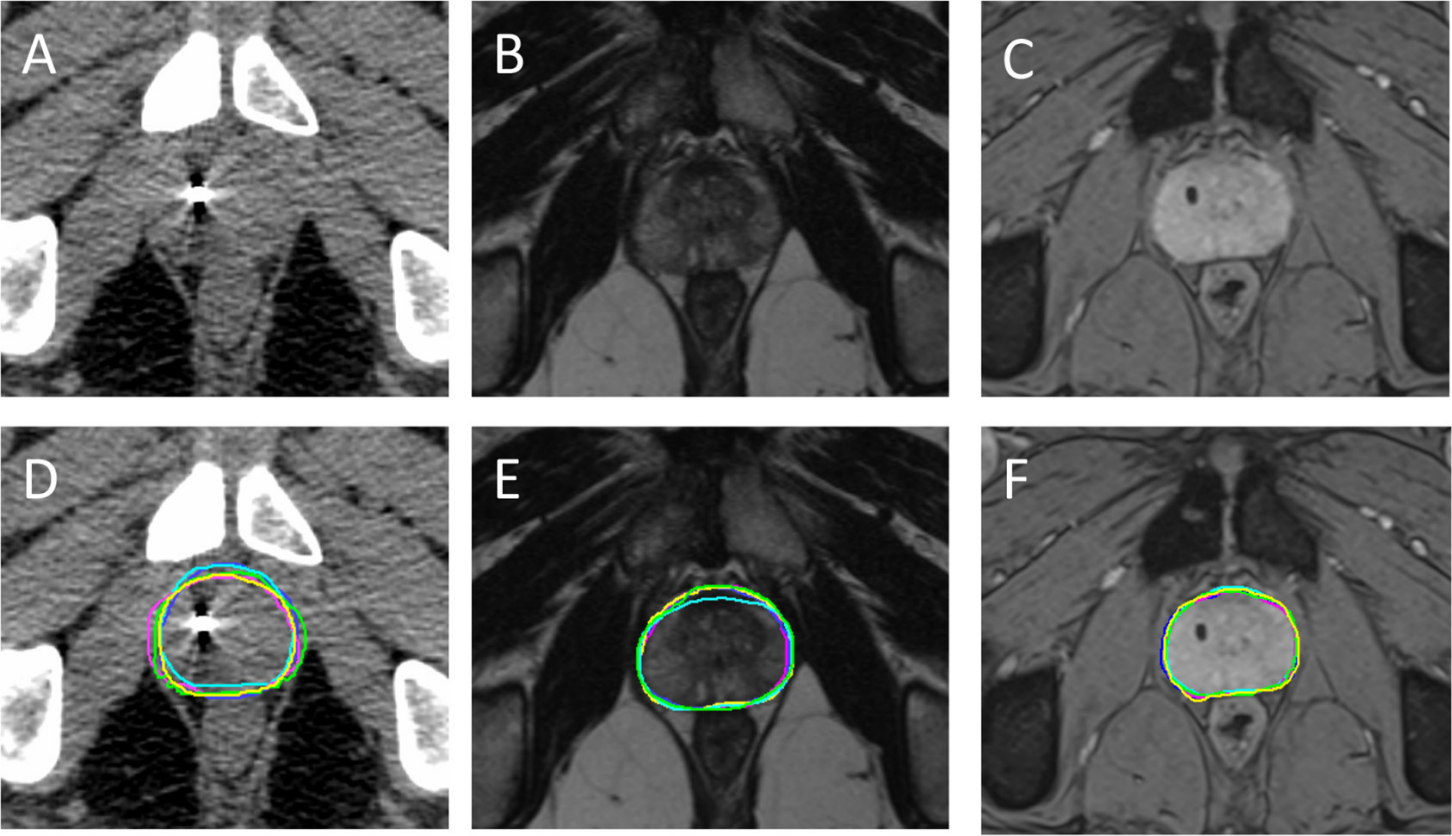
A–C are examples of CT, *T*_2_W and *T*_2_*W imaging at corresponding levels for the same patient, without contours. D–F demonstrate the same imaging with superimposed radiographer contours. Reproduced from published abstract format with permission.^13^

At least 1 week prior to planning imaging, three 1.0 × 3.0 mm knurled gold fiducial markers are inserted under transrectal ultrasound guidance. Patients are instructed regarding bladder filling and rectal preparation as per departmental guidelines prior to their imaging sessions. The latter consists of two days of rectal preparation with microenemas prior to their CT planning appointment, and an enema just before their planning CT scan. The treatment set-up position is replicated for planning imaging. The CT extends in 1.5 mm axial slices from the mid-lumbar spine to below the obturator foramen. This is followed, on the same day, by the planning MRI scan at 1.5 T (Siemens Aera, Erlangen, Germany) with 2 two-dimensional sequences, covering the prostate volume using 28 adjacent slices (at 2.5 mm thickness). Firstly, a standard *T*_2_W pulse sequence used in diagnostic prostate MRI and based on fast spin echoes, allowing visualisation of the internal structure of the prostate, is acquired. The second sequence is *T*_2_*W combining several gradient echo signals, with a range of echo-times into a single image, thereby maximising the signal loss related to the fiducial markers. Examples of images are shown in Figure 1.

### Contouring

The contouring and analysis methods have previously been described in published abstract format.^13^ Three clinicians experienced in prostate RT delineated the prostate on each of the three imaging data sets—CT, *T*_2_W and *T*_2_*W MRI using Monaco v.5.19.02 (research version, Elekta AB, Stockholm, Sweden). All contours were created individually, without reference to other types of imaging. A minimum of 2 weeks was left between contouring images of the same patient to avoid recall bias. A simultaneous truth and performance level estimate (STAPLE)^14^ contour was created from all three clinician contours for each imaging set to create the "gold-standard" for comparison. The interobserver variability for these clinician contours has previously been reported with median Dice similarity co-efficient (DSC) of 0.95 (interquartile range 0.94–0.96), 0.97 (0.96–0.97) and 0.97 (0.96–0.97) for CT, *T*_2_W and *T*_2_*W imaging respectively.^9^

Five therapeutic radiographers experienced in delineation and/or registration of the prostate on CT and CBCT, completed a single training session, delivered by a clinical oncologist. The training included review of the anatomy on each of the three imaging types and access to CT, *T*_2_W and *T*_2_*W “atlases” with axial contours to refer to. The radiographers then delineated the prostate on CT, *T*_2_W and *T*_2_*W MRI for the same 10 patients using the same instructions. In addition, the time taken for delineation was recorded and images were scored from 0 to 10 for “image quality” and “confidence in contouring”, where a higher score indicates an improvement.

### Analysis of contours

Assessment was made of;

1. *﻿Interobserver variability*—a STAPLE contour was created from the contours of all five radiographers. Each individual contour was then compared to this STAPLE contour to assess radiographer interobserver variability.
2. *Accuracy—*﻿by comparison of radiographer contours to the gold standard’ clinician STAPLE.

Contours were assessed using Monaco ADMIRE software v.2.0 (research version, Elekta AB, Stockholm, Sweden). For each comparison, the overlap measures DSC and Cohen’s kappa (κ) were recorded, (where higher values indicate greater agreement). In addition, the distance measures of Hausdorff distance and mean distance between contours were recorded (where lower values indicate greater agreement).

Using GraphPad Prism v7.0d, non-parametric Friedman testing was performed with Dunn’s test for multiple comparisons. The three imaging comparisons—CT *vs.* *T*_2_W, CT *vs.* *T*_2_*W and *T*_2_W *vs.* *T*_2_*W were pre-planned. Values were defined as statistically different if the adjusted *p*-value was <0.05.

## Results

Examples of radiographer contours are shown in Figure 1, reproduced from published abstract format with permission.^13^

**Figure 2.**
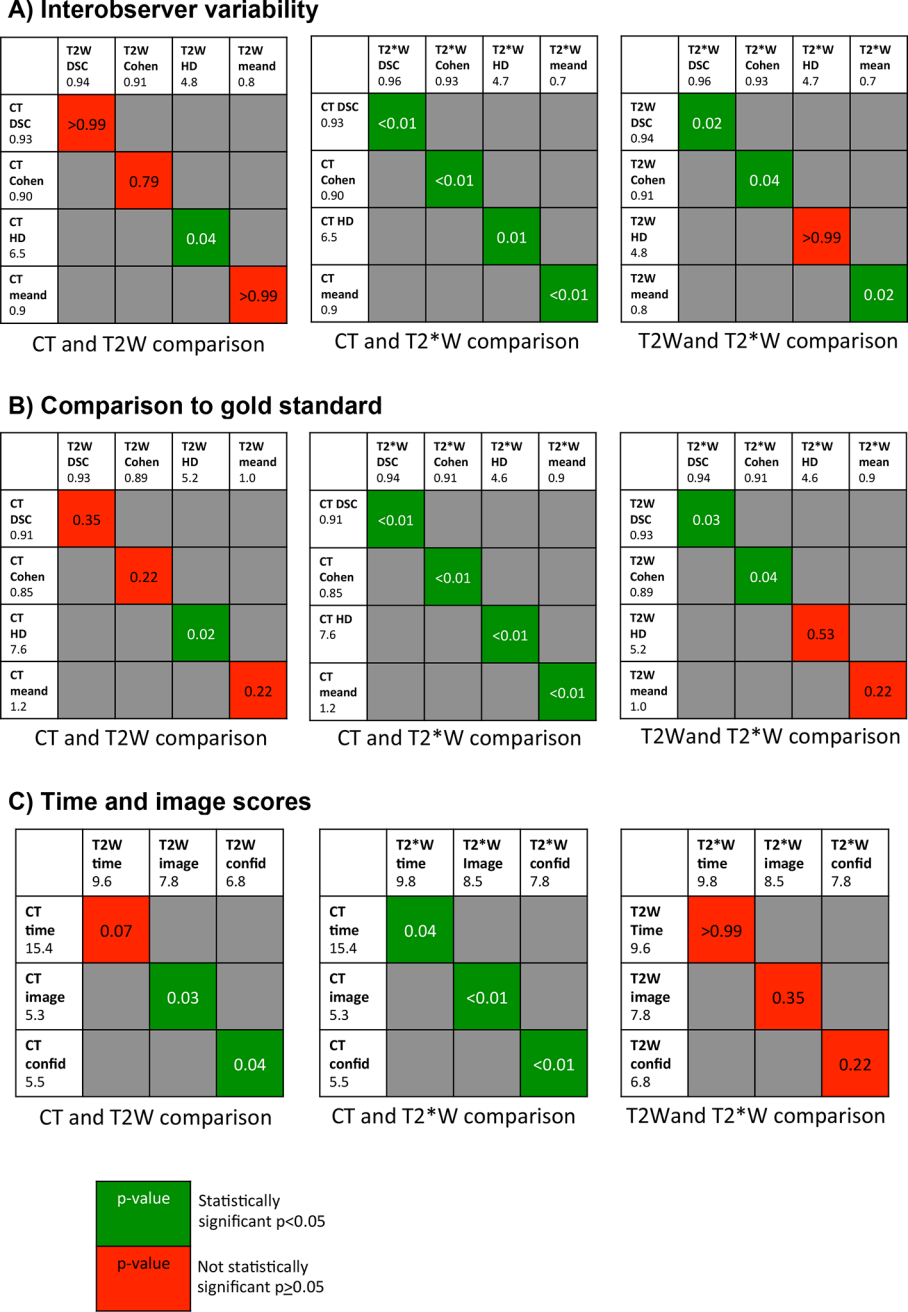
Summary of *p*-values (reported to two decimal places) from statistical testing for comparison between imaging modalities. Values are adjusted for multiple comparisons and statistically significant if p<0.05. Abbreviations: Cohen,Cohen’s κ; mean d, mean distance between contours; confid, confidence in contouring score; image, image quality score; .

Median (interquartile range) comparisons for each imaging type, delineation times and imaging scores are summarised in Table 1. Results of statistical testing are summarised in Figure 2.

**Table 1.** Summary of median (interquartile range) comparison values for each imaging type

		**CT**	***T*_2_W MRI**	*T*_2_*W MRI
Interobserver variability	DSC	0.93 (0.91–0.95)	0.94 (0.93–0.95)	0.96 (0.95–0.96)
	Cohen κ	0.90 (0.87–0.91)	0.91 (0.89–0.92)	0.93 (0.92–0.94)
	HD (mm)	6.5 (5.7–7.9)	4.8 (4.2–5.8)	4.7 (3.9–5.4)
	Mean d (mm)	0.9 (0.8–1.1)	0.8 (0.7–1.0)	0.7 (0.6–0.7)
Comparison to gold-standard	DSC	0.91 (0.89–0.92)	0.93 (0.91–0.94)	0.94 (0.93–0.95)
	Cohen κ	0.85 (0.83–0.88)	0.89 (0.86–0.90)	0.91 (0.89–0.93)
	HD (mm)	7.6 (6.6–9.1)	5.2 (4.4–6.2)	4.6 (4.0–5.5)
	Mean d (mm)	1.2 (1.2–1.4)	1.0 (0.9–1.2)	0.9 (0.7–1.0)
Assessment of contouring efficiency	Time taken to contour (min)	15.4 (12.0–16.3)	9.6 (8.3–12.6)	9.8 (8.9–10.9)
	Image quality (0–10)	5.3 (5.2–5.8)	7.8 (7.4–8.1)	8.5 (8.2–8.8)
	Confidence in contour (0–10)	5.5 (5.2–5.6)	6.8 (6.7–7.3)	7.8 (7.5–7.9)

DSC, Dice similarity co-efficient; HD, Hausdorff distance; d, distance.

Values are reported to one decimal place apart from overlap measures reported to two decimal places.

The high overlap values, with all DSC and Cohen’s κ >0.85, illustrate the good agreement between radiographers and between radiographers and the gold-standard across all imaging types.

On comparison of MRI to CT, both *T*_2_W and *T*_2_*W contours show higher overlap values and lower distance values, indicating reduced interobserver variability and improved accuracy when compared to the gold standard. This was statistically significant for *T*_2_*W contours compared to CT across all four comparison metrics.

In addition, comparison of the two MRI sequences reveals that prostate contours delineated using *T*_2_*W MRI show significantly decreased interobserver variability for all measurements excluding Hausdorff distance, and significantly improved accuracy for DSC and Cohen’s κ when compared to *T*_2_W MRI. (Table 1/ Figure 2).

Greater quality images and confidence in contouring were reported for both MRI types but especially *T*_2_*W MRI, reflected in the shorter time to complete contours, with a median of 9.6–9.8 min for MRI compared to 15.4 min for CT.

## Discussion

We have demonstrated that despite the unfamiliarity of MRI, interobserver variability and accuracy of therapeutic radiographer prostate contours improved with both MRI sequences, in particular the *T*_2_*W sequence.

We have considered both consistency and accuracy of contours. The reduced interobserver variability on MRI is in keeping with previous results from clinician contouring^15–17^ as a result of improved soft tissue contrast, reflected in the higher scores for image quality and confidence in contouring. However, this was only statistically significant across all four measures for *T*_2_*W *vs.* CT.

For accuracy of contouring, a gold-standard for RT planning is difficult to define; here we have used the STAPLE of three experienced clinicians to reduce the effect of interobserver variability for the gold-standard contour. All observers, both clinicians and radiographers, are from the same institution, which will influence both consistency and accuracy, as assessed by the overlap and distance measurements here and previously.^9^

With regards to time, the prostate was delineated on both MR sequences more quickly compared to CT. There was a reduction in the median time for contouring by 5.6 and 5.8 min for *T*_2_*W and *T*_2_W MRI respectively. This is particularly relevant for contouring in an online adaptive workflow, where shortening this step is beneficial to minimise intrafractional motion. Although the time improvement with MRI is mirrored in the higher confidence in contouring and image quality of MRI compared to CT, note must be made of the differing slice thickness of the images—1.5 mm for CT and 2.5 mm for MRI. As a result, there were a greater number of slices over the length of the prostate for contouring on CT compared to MRI. Although observers were allowed to use interpolation of contours if desired on any of the image sets, the time taken must be interpreted with caution for this reason.

There is no consensus on the best method for contour comparison,^18,19^ we have therefore used a combination of comparison values here to encompass the overlap and distance between contours. Although we have carried out statistical testing here, we have not assessed the clinical impact of a significant difference in these comparisons. For example, the clinical implication of a DSC of 0.93 *vs* 0.95 may be negligible although this will also be dependent on where the discrepancy lies and the margins added during planning. The resulting dosimetric effect, not assessed here, would be more relevant.^20^

Our findings are particularly important as we have commenced MR-guided RT at the Royal Marsden Hospital with daily online replanning, which requires recontouring on images acquired each day. The process either involves manual contouring from the beginning or amending propagated contours produced by deformable registration of the reference image to the new daily acquired image. To begin with, this is clinician led with the aim of expanding the role of our radiographers to encompass this step. This is an essential progression of the extended role which has developed from evaluating treatment portal images,^21^ evaluating verification images for hypofractionated treatments,^22^ and to more recently, choosing the “plan of the day”.^23^ Accurate target identification is also required for motion monitoring of the target prostate during treatment delivery. Contributing to current literature, our study has considered the practical points of “confidence in contouring” and the time taken, both highly relevant in the time pressured online adaptive RT setting.

Most relevant literature to date makes use of *T*_2_W images which are the mainstay of MRI for diagnosis and staging. We have proposed the *T*_2_*W sequence, which not only allows visualisation of the fiducials, particularly important for a mixed CT-MR workflow, but also provides improved contrast between the prostate and surrounding tissues. MRI for delineation is not used routinely outside of a trial setting in our institution but implanted fiducial markers are used for image guidance prior to each fraction. Our study shows that sequences such as *T*_2_*W MRI, allowing improved prostate capsule visualisation and contour accuracy, can continue to be useful even if fiducials are no longer required, such as with the clinical use of MR only workflow.

The work we have presented here is novel, in addition to establishing the accuracy and consistency of contours for this professional group, we have demonstrated the relevance of sequence selection and validated the use of the *T*_2_*W sequence. Our work will be expanded further to assess the dosimetric impact of any differences in contours and consider the use of the *T*_2_*W sequence for automatic contouring. A formal training programme will also be designed for therapeutic radiographer training as the role of MR-guided RT develops.

## Conclusions

Despite unfamiliarity with MRI for treatment verification, therapeutic radiographer prostate contours are more accurate, show less interobserver variability and are more confidently and quickly outlined on MRI compared to CT. In addition, this improvement is consistently statistically significant for the *T*_2_*W MRI sequence. This is particularly relevant for MRI sequence choice and development of the roles of the interprofessional team in the advancement of MRI-guided RT.
